# Food-breastmilk combinations alter the colonic microbiome of weaning infants: an *in silico* study

**DOI:** 10.1128/msystems.00577-24

**Published:** 2024-08-27

**Authors:** Vitor G. da Silva, Nick W. Smith, Jane A. Mullaney, Clare Wall, Nicole C. Roy, Warren C. McNabb

**Affiliations:** 1 Riddet Institute, Massey University, Palmerston North, New Zealand; 2 High-Value Nutrition National Science Challenge, Auckland, New Zealand; 3 AgResearch, Palmerston North, New Zealand; 4 Department of Nutrition and Dietetics, The University of Auckland, Auckland, New Zealand; 5 Department of Human Nutrition, University of Otago, Dunedin, New Zealand; Pontificia Universidad Catolica de Chile, Santiago, Santiago, Chile

**Keywords:** gut microbiome, food, infant, *in silico*

## Abstract

The introduction of solid foods to infants, also known as weaning, is a critical point for the development of the complex microbial community inhabiting the human colon, impacting host physiology in infancy and later in life. This research investigated *in silico* the impact of food-breastmilk combinations on growth and metabolite production by colonic microbes of New Zealand weaning infants using the metagenome-scale metabolic model named Microbial Community. Eighty-nine foods were individually combined with breastmilk, and the 12 combinations with the strongest influence on the microbial production of short-chain fatty acids (SCFAs) and branched-chain fatty acids (BCFAs) were identified. Fiber-rich and polyphenol-rich foods, like pumpkin and blackcurrant, resulted in the greatest increase in predicted fluxes of total SCFAs and individual fluxes of propionate and acetate when combined, respectively, with breastmilk. Identified foods were further combined with other foods and breastmilk, resulting in 66 multiple food-breastmilk combinations. These combinations altered *in silico* the impact of individual foods on the microbial production of SCFAs and BCFAs, suggesting that the interaction between the dietary compounds composing a meal is the key factor influencing colonic microbes. Blackcurrant combined with other foods and breastmilk promoted the greatest increase in the production of acetate and total SCFAs, while pork combined with other foods and breastmilk decreased the production of total BCFAs.

**IMPORTANCE:**

Little is known about the influence of complementary foods on the colonic microbiome of weaning infants. Traditional *in vitro* and *in vivo* microbiome methods are limited by their resource-consuming concerns. Modeling approaches represent a promising complementary tool to provide insights into the behavior of microbial communities. This study evaluated how foods combined with other foods and human milk affect the production of short-chain fatty acids and branched-chain fatty acids by colonic microbes of weaning infants using a rapid and inexpensive *in silico* approach. Foods and food combinations identified here are candidates for future experimental investigations, helping to fill a crucial knowledge gap in infant nutrition.

## INTRODUCTION

The human gastrointestinal tract is colonized by a complex microbial community, with the greatest concentration and diversity being found in the large intestine, or colon (1). Diet is a well-known key factor shaping the composition and function of colonic microbes throughout human life (2–4). In turn, colonic microbes impact host physiology and are associated with health and disease biomarkers (5). One mechanism by which the colonic microbiota affects host health is the production of metabolites that are later absorbed by the host (6). Organic acids, such as short-chain fatty acids (SCFAs) and branched-chain fatty acids (BCFAs), are among the most studied microbial metabolites produced in the colon, and decreases in their production have been associated with disease, particularly gastrointestinal and metabolic disorders (7, 8).

One of the challenges of investigating the colonic microbiome is that the composition of this microbial community is unique to each individual, making it difficult to define an ideal microbiota in terms of composition (9, 10). On the other hand, the functional capacity of the microbiota is similar among individuals with the same health status (11), and colonic microbes are functionally redundant, meaning that different taxa can perform the same metabolic functions (12). Thus, focusing on what the colonic microbiota produces, rather than which microbes compose it, may be more relevant to host health implications, also reducing the complexity of microbiome investigations.

Animal and human studies have demonstrated that imbalances in the colonic microbiota, or dysbiosis, during infancy are associated with an increased risk for several diseases later in life (13–16). Therefore, adequate nutrition from an early postnatal age is crucial to prevent or limit dysbiosis. The introduction of solid foods represents a window of opportunity for establishing long-term beneficial host-microbiota interactions that may influence host physiology later in life (17, 18). However, among the studies that examined the impact of solid foods on the colonic microbiota of infants (19–24), only a few assessed the microbial production of organic acids (25, 26), limiting our understanding of how complementary foods affect colonic microbes in this crucial life stage.

Studies conducted *in vitro* or using animal models have investigated the effect of foods on the colonic microbiome of infants in the absence of human milk (27–30), not accurately reflecting how complementary foods are introduced to weaning infants. Furthermore, there is no evidence *in vitro*, in animals (such as mice and piglets), or *in silico* of how food-breastmilk combinations affect the colonic microbiota in early life.

Mathematical models are a rapid and inexpensive complementary strategy to study microbial communities, being able to generate hypotheses that can be further tested by experimental approaches. Genome-scale metabolic models stand out for using genome information to infer the metabolism of microorganisms, having the major advantage of predicting microbial growth rates and fluxes of produced metabolites without prior definition of kinetic parameters, whose experimental determination is technically challenging (31).

Recently, a metagenome-scale community metabolic model named Microbial Community (MICOM) was proposed to predict how diets shape the composition and function of the human colonic microbiota (29). So far, *in silico* investigations of the colonic microbiome using metagenome-scale metabolic models have mainly focused on adults (32–35). This study used this modelling approach to identify foods with the strongest impact on the production of organic acids by colonic microbes of weaning infants when combined with breastmilk. SCFAs and BCFAs were chosen due to their association with the host health. Insights generated by this research help the design of future experiments, advancing the understanding of the relationship between complementary foods and colonic microbes in a decisive stage of human life.

## RESULTS

### Predicted fluxes of SCFAs and BCFAs

This study aimed to verify *in silico* the effect of complementary foods on the colonic microbiota of New Zealand weaning infants. For that, a literature survey was conducted to identify 89 foods from various food groups that are commonly introduced to weaning infants, particularly in New Zealand (36–40). These foods were combined *in silico* with breastmilk to create combinations representative of those consumed by 6-month-old infants (15% food and 85% breastmilk, by caloric intake). We further evaluated *in silico* the effect of these food-breastmilk combinations on the 10 most abundant genera composing the average fecal microbiota of New Zealand infants at weaning age (5–12 months of age), using previously published data (28). Candidate foods with the strongest impact on the microbial production of organic acids (SCFAs and BCFAs) were identified. These candidate foods were chosen to provide a diversity of model outcomes, including foods that increased, decreased, or had little effect in total SCFAs (acetate, propionate, and butyrate combined) or total BCFAs (isobutyrate and isovalerate combined) fluxes compared to breastmilk alone.

**Fig 1:**
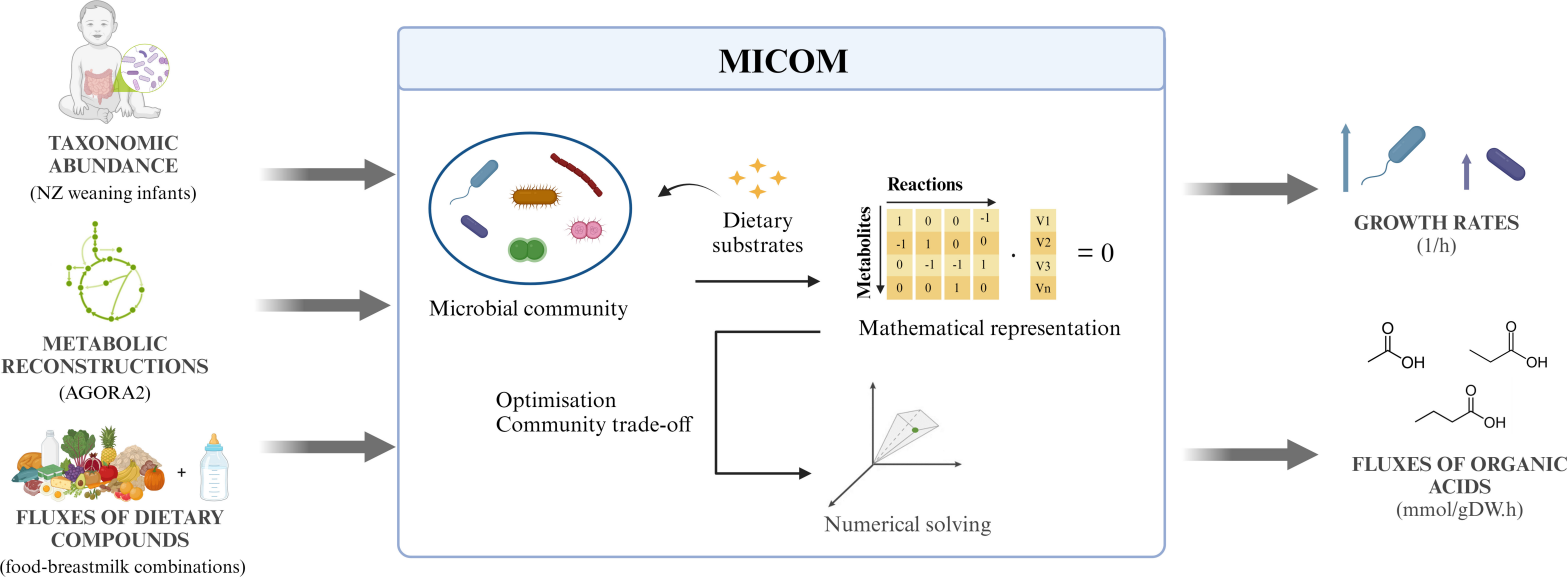
Workflow to predict the influence of food-breastmilk combinations on growth rates of colonic microbes of weaning infants and produced fluxes of organic acids using the model MICOM. Image created using Biorender.com.

Importantly, the employed modeling approach results in repeatable outcomes when simulations are performed with the same initial conditions. Consequently, statistical analyses could not be performed to assess any potential variation in microbial growth or metabolite production resulting from the use of different food-breastmilk combinations. Another limitation of this study was using a 1% relative abundance threshold to select the microbial genera included in the simulations, resulting in a small-scale community that lacked low-abundant taxa and may not fully represent the metabolic potential of the colonic microbiota of weaning infants. Thus, the findings presented are preliminary and require further experimental validation. A graphical representation of the modeling workflow is depicted in Fig. 1.

**Fig 2:**
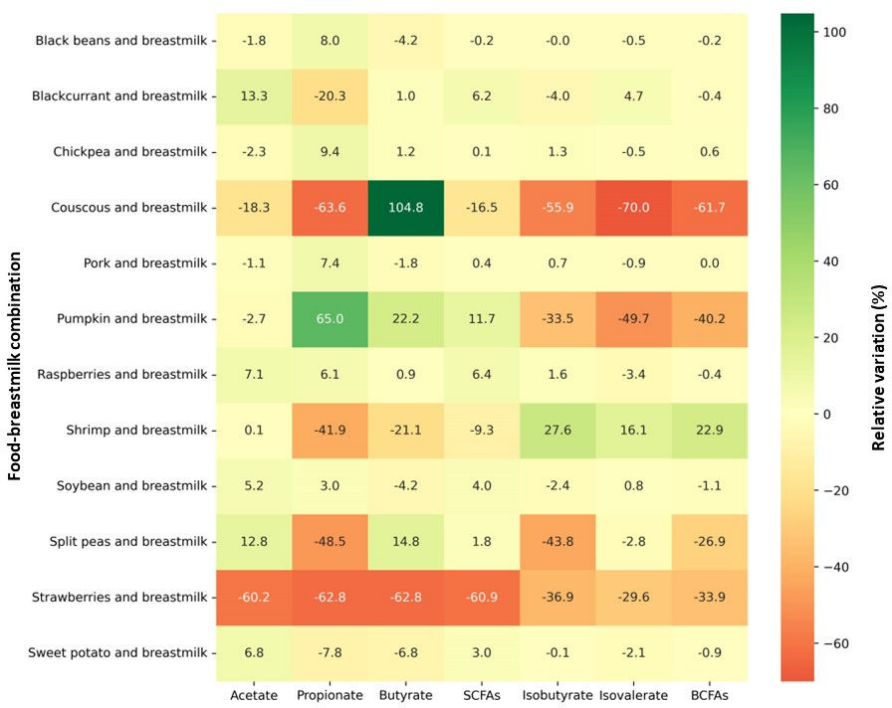
Heatmap of food-breastmilk combinations with the greatest influence on predicted fluxes of SCFAs and BCFAs. Fluxes of organic acids are expressed in relative variation in comparison to breastmilk alone. Cells are colored according to intensity, with the highest values in green and the lowest values in red.

Combining foods with breastmilk altered the fluxes of major SCFAs and BCFAs compared to breastmilk alone (Table S1). Twenty-nine food-breastmilk combinations increased the flux of total SCFAs (32% of the evaluated combinations), and 64 food-breastmilk combinations reduced the flux of total BCFAs (72% of the combinations). Twelve food-breastmilk combinations that, compared to breastmilk alone, resulted in the greatest increase, decrease, or similar values of total SCFAs and BCFAs fluxes were identified (Fig. 2).

The combination of pumpkin with breastmilk promoted the greatest increase in the flux of total SCFAs (11.7%) and the second greatest decrease in the flux of total BCFAs (40.2%), compared to breastmilk alone. Raspberries-breastmilk and blackcurrant-breastmilk increased the production of total SCFAs (6.4 and 6.2%, respectively) with no change in the flux of total BCFAs. Similarly, soybean-breastmilk and sweet potato-breastmilk elevated fluxes of total SCFAs (4.0 and 3.0%, respectively) without changing total BCFA production.

The increase in the flux of total SCFAs obtained with the pumpkin-breastmilk combination was driven by heightened fluxes of propionate and butyrate. On the other hand, the other four food-breastmilk combinations increasing total SCFA flux increased the flux of acetate. Indeed, combining pumpkin with breastmilk resulted in the largest increase in propionate (65.0%), while breastmilk combined with blackcurrant in the largest increase in acetate (13.3%).

The couscous-breastmilk combination resulted in the highest increase in butyrate (104.8%) but decreased acetate and propionate individually. In addition, it caused the largest reduction in total BCFA flux (61.7%). Split peas-breastmilk reduced the flux of total BCFAs by 26.9% while increasing the production of acetate (12.8%) and butyrate (14.8%). On the other hand, the combination of strawberry with breastmilk promoted the greatest reduction in the total SCFA flux (60.9%) also reducing total BCFA flux (33.9%), while shrimp-breastmilk had the greatest increase in the production of BCFAs (22.9%). Conversely, black beans, pork, and chickpeas promoted little to no change in the fluxes of SCFAs and BCFAs when individually combined with breastmilk.

### Predicted microbial growth rates

The modeling approach makes predictions about microbial growth assuming that the system is in a steady state, meaning that there is no accumulation of substrates. In addition, MICOM employs a linearization strategy that correlates the predicted growth rates of individual taxa to their relative abundance. As a result, the profile of faster-growing microbes was not expected to change much under different food-breastmilk combinations. High-abundance genera were expected to have the greatest growth rates, while low-abundance genera were expected to have slower growth rates.

As expected, the high-abundance genera *Bifidobacterium*, *Bacteroides*, and *Bacillus* had higher growth rates for most of the food-breastmilk combinations, while low-abundance genera, such as *Lacticaseibacillus* and *Streptococcus*, had slower growth rates (Table S2). On the other hand, low-abundance genera also had negligible growth (growth rates lower than 10^−6^). This result suggests a failure to respect the trade-off between community growth and individual growth, resulting in a numerical problem that was not optimally solved (numerical instabilities, see Materials and Methods section).

**Fig 3:**
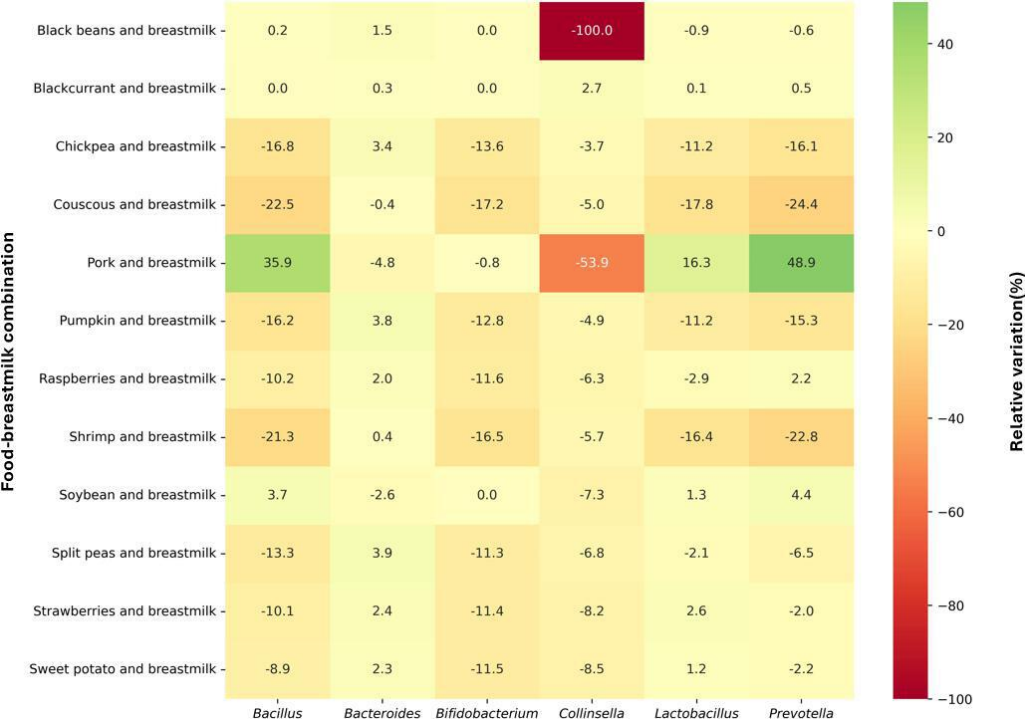
Heatmap of microbial growth rates for food-breastmilk combinations with the greatest influence on predicted fluxes of SCFAs and BCFAs. Microbial growth rates are expressed in relative variation in comparison to breastmilk alone. Cells are colored according to intensity, with the highest values in green and the lowest values in red. Genera with predicted negligible growth are not presented.

Food-breastmilk combinations with the greatest influence on the fluxes of SCFAs and BCFAs impacted the microbial growth rates of high-abundance genera in different ways (Fig. 3). Compared to breastmilk alone, little to no changes in the growth rates of the genus *Bacteroides* were observed for these breastmilk-food combinations. On the other hand, most of the food-breastmilk combinations reduced the growth of the *Bifidobacterium* and *Lactobacillus* genera. The greatest reduction in these genera was observed for couscous-breastmilk (17% reduction), followed by shrimp-breastmilk (16%). In addition, a few food-breastmilk combinations altered the growth of *Prevotella*, *Collinsella*, and *Bacillus* genera. The pork-breastmilk combination had the strongest influence on the growth rates of these genera, increasing it by 48% for the *Prevotella* genus and 35% for the *Bacillus* genus, while decreasing growth rates by 53% for the *Collinsella* genus.

### Impact of multiple food-breastmilk combinations on colonic microbes of weaning infants

As complementary foods are normally introduced to infants in combination with other foods rather than consumed individually, the identified foods with the strongest impact on microbial SCFAs and BCFAs fluxes were combined with other identified foods and breastmilk for additional simulations. Sixty-six combinations were generated (multiple food-breastmilk combinations), composed of 7.5% of a first food item, 7.5% of a second food item, and 85% of breastmilk by caloric intake. In comparison to breastmilk alone, 24 multiple food-breastmilk combinations increased the flux of total SCFAs (36% of the combinations), while 47 reduced the flux of total BCFAs (70% of the combinations, Table S3).

**Fig 4:**
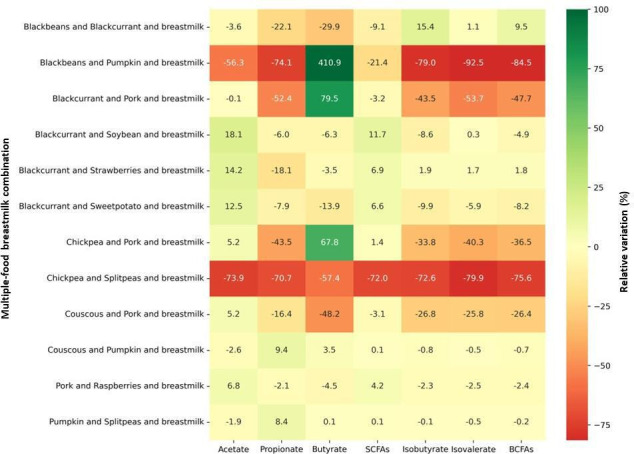
Heatmap of multiple food-breastmilk combinations with the greatest influence on predicted fluxes of SCFAs and BCFAs. Fluxes of organic acids are expressed in relative variation in comparison to breastmilk alone. Cells are colored according to intensity, with the highest values in green and the lowest values in red.

The combination of blackcurrant with breastmilk and soybean, strawberries, or sweet potato promoted the highest increases in acetate production, leading to the greatest increases in total SCFA flux by 11.7%, 6.9%, and 6.6%, respectively, as compared to breastmilk (Fig. 4). Pumpkin combined with breastmilk and couscous, or split peas resulted in the greatest increase in propionate (9.4% and 8.4%, respectively) but did not alter total SCFA flux. On the other hand, the combination of black beans-pumpkin-breastmilk had the greatest increase in butyrate flux (410.9%).

However, the same combination had the greatest reduction in isobutyrate, isovalerate, and resulting total BCFA flux (84.5%). The combination chickpea-split peas-breastmilk followed behind, with a total BCFA flux reduction of 75.6%. Pork combined with breastmilk and other foods also decreased total flux of BCFAs; reduction was observed for the pork-couscous-breastmilk (26.4%), pork-chickpea-breastmilk (36.5%), and pork-blackcurrant-breastmilk combinations (47.7%).

**Fig 5:**
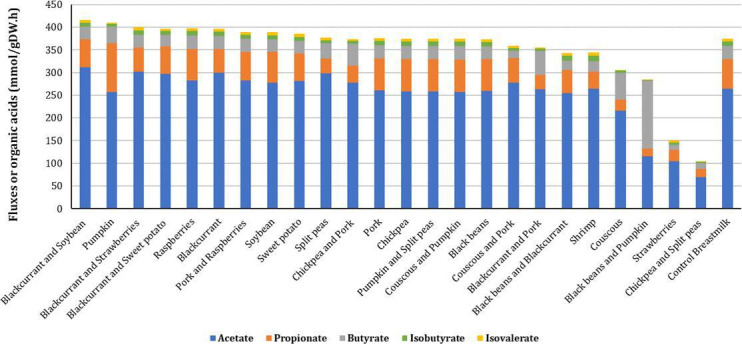
Comparison of SCFA and BCFA fluxes when foods are combined individually with breastmilk or with other foods and breastmilk. Food-breastmilk combinations are ordered according to their predicted total SCFA flux, in which combinations resulting in higher fluxes are shown on the left. Fluxes for breastmilk alone (control) are shown on the right.

Combining foods with other foods and breastmilk shifted the way foods influenced the production of total SCFAs and total BCFAs when combined with breastmilk only. Among the foods individually combined with breastmilk, pumpkin promoted the greatest increase in the flux of total SCFAs, while combining pumpkin with other foods and breastmilk, such as pumpkin-split peas-breastmilk and couscous-pumpkin-breastmilk, promoted little to no changes in the flux of total SCFAs (Fig. 5). In turn, among the multiple food-breastmilk combinations, it was blackcurrant that stood out for promoting the greatest increases in total SCFA flux when combined with breastmilk and soybean, strawberries, or sweet potato. Most concerning, the combination of strawberries-breastmilk promoted the smallest total SCFA flux, but when strawberries were combined with blackcurrant and breastmilk, it resulted in the second highest increase in total SCFA flux (Fig. 5).

Nevertheless, the influence of pumpkin and blackcurrant on the production of propionate and acetate, respectively, was maintained when combining those foods with other foods and breastmilk. Although the combinations of couscous-pumpkin-breastmilk and pumpkin-split peas-breastmilk did not change total SCFA flux, they resulted in the greatest increase in propionate. Similarly, the combinations of blackcurrant-soybean-breastmilk, blackcurrant-strawberries-breastmilk, and blackcurrant-sweet potato-breastmilk had the highest increases in acetate flux.

As expected, high-abundance genera, like *Bifidobacterium*, *Bacteroides*, and *Bacillus*, grew faster, while low-abundance genera, such as *Lacticaseibacillus* and *Streptococcus*, grew slower under multiple food-breastmilk combinations (Table S4). Negligible growth (growth rates near zero) for the low-abundance genera was also observed. For instance, 55 out of 89 multiple-food breastmilk combinations did not promote the growth of at least one genus included in the simulations, indicating that the trade-off between maximal community growth and individual growth was not respected, probably due to numerical issues.

**Fig 6:**
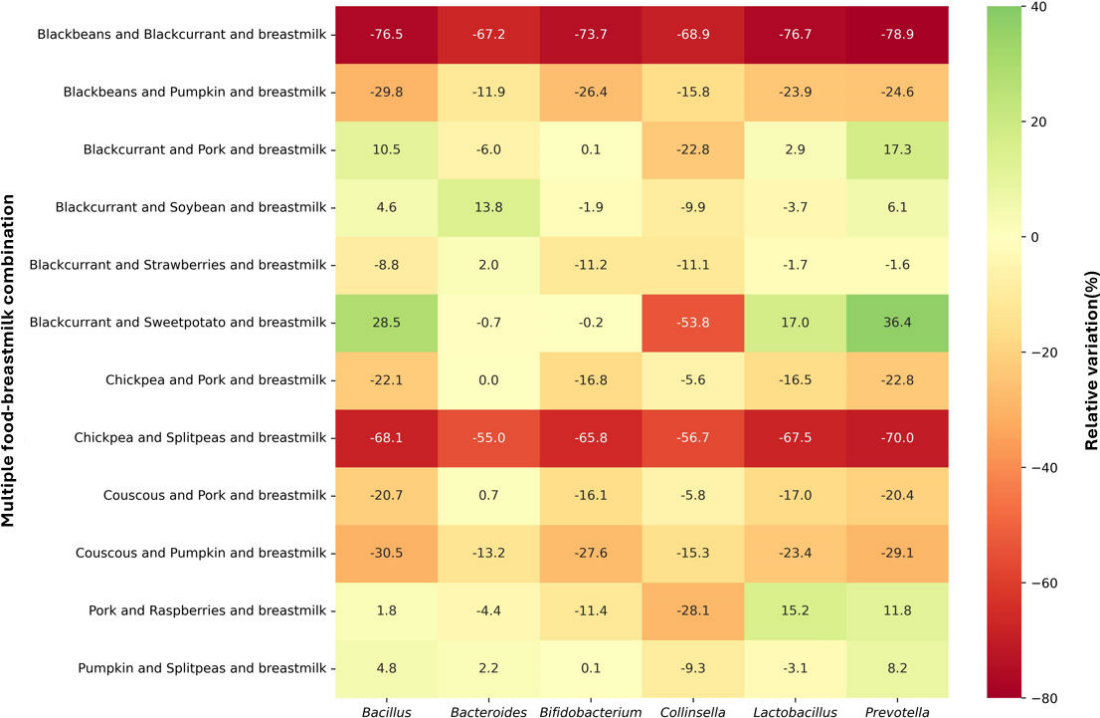
Heatmap of microbial growth rates for multiple food-breastmilk combinations with the greatest influence on predicted fluxes of SCFAs and BCFAs. Microbial growth rates are expressed in relative variation in comparison to breastmilk alone. Cells are colored according to intensity, with the highest values in green and the lowest values in red. Genera with predicted negligible growth are not presented.

Overall, multiple food-breastmilk combinations tended toward decreasing the growth of the *Bifidobacterium* and *Lactobacillus* genera. Among the combinations with the greatest influence on the production of total SCFAs and BCFAs, blackcurrant-soybean-breastmilk resulted in the greatest increase in the growth rates of the *Bacteroides* genus (13.8%, Fig. 6). Blackcurrant-sweet potato-breastmilk promoted the greatest relative increases in the growth rates of the genera *Prevotella* (36.4%), *Bacillus* (28.5%), and *Lactobacillus* (17.0%), while black beans-blackcurrant-breastmilk the greatest relative decreases for these same genera (78.9, 76.5, and 76.7%, respectively). On the other hand, the combinations of chickpea-split peas-breastmilk, black beans-blackcurrant-breastmilk, and black beans-pumpkin-breastmilk decreased the growth of all high-abundance genera.

## DISCUSSION

In this study, a metagenome-scale metabolic modeling approach was employed to identify foods and food combinations with the greatest impact on total SCFA and BCFA production by the colonic microbiota of New Zealand weaning infants. This is the first *in silico* screening of the influence of complementary foods on the colonic microbes of weaning infants. Another originality of this study was the *in silico* evaluation of foods combined with breastmilk and other foods, allowing a better representation of how complementary foods are introduced to weaning infants.

A total of 155 food-breastmilk combinations (89 single food-breastmilk and 66 multiple-food breastmilk combinations) were investigated, and the 12 individual foods and 12 food combinations with the strongest influence on predicted fluxes of total SCFAs and BCFAs, when combined with breastmilk, were identified. Consistent with nutritional recommendations for weaning infants (41), foods included in the simulations were from diverse food groups, including legumes, fruits, vegetables, and animal proteins, while sweets and fast-food products were excluded. For instance, legumes and meats are recommended for infants as sources of protein and iron, while fresh fruits and vegetables are sources of certain vitamins, other minerals, and dietary fiber (39, 41, 42).

MICOM assumes a steady state and employs a two-step linearization strategy that predicts growth rates based on the relative abundance of individual taxa (32). Furthermore, the food-breastmilk combinations consisted of 85% breastmilk by caloric intake, resulting in a similar nutritional profile. As a result, the predicted profile of faster-growing microbes was expected to remain consistent across different food-breastmilk combinations, with high-abundance genera having higher growth rates than low-abundance genera. As expected, the highest growth rates were observed for highly abundant genera, while less abundant genera had slower growth. However, predicted microbial growth was not homogeneous under different dietary conditions, and growth rates near zero were also predicted for the less abundant genera *Limosilactobacillus*, *Lacticaseibacillus*, *Streptococcus*, and *Veillonella*. This indicates that the solver failed to optimally solve the linear problem, most likely affecting outcomes of predicted growth rates and fluxes of organic acids.

Compared to breastmilk (control), most of the food-breastmilk combinations decreased the growth rate of the *Bifidobacterium* and *Collinsella* genera, agreeing with findings from longitudinal studies showing the positive association between breastmilk consumption and the relative abundance of these genera (2, 17, 20). On the other hand, only small increases (up to 3.9%) in the growth rate of the genus *Bacteroides*, which are associated with complex carbohydrates and protein degradation, were predicted *in silico*. This contrasts with the observed increased fecal relative abundance of the *Bacteroides* genus when solid foods are introduced to infants (3, 43).

Nevertheless, our ability to compare *in silico* predictions of microbial growth rates with *in vitro* or *in vivo* observed microbial abundance is limited. It is a technical challenge to accurately measure the growth rates of individual colonic microbes experimentally (44) and available data are rare, particularly for under-investigated groups like weaning infants. Furthermore, changes in microbiota composition are not as informative as changes in microbiota function, considering that healthy individuals tend to have distinct microbiota composition but with similar functionality (9, 11). Thus, the focus of the study was on how food-breastmilk combinations affect the microbial production of health-relevant organic acids, particularly SCFAs and BCFAs, in the colonic microbiota of infants, rather than its microbial composition.

As compared to breastmilk alone, pumpkin, raspberries, blackcurrant, soybeans, and sweet potato individually combined with breastmilk resulted in the greatest increases in the flux of total SCFAs. Among these foods, pumpkin and blackcurrant also stood out for promoting the greatest increase in the production of propionate and acetate, respectively. These findings suggest beneficial alterations to the colonic microbiome of infants at weaning, as SCFAs are associated with health benefits (45–48).

SCFAs are primarily produced in the colon through the microbial fermentation of non-absorbed carbohydrates like dietary fiber and resistant starch. Acetate is the most abundant SCFA, especially during the first months of life, having anti-inflammatory properties and contributing to epithelial barrier integrity (49, 50). As the colonic microbiota matures during the weaning period, the proportion of propionate and butyrate in the colon increases (49). These organic acids, in addition to supporting colonic barrier integrity, provide various other health benefits, such as promoting metabolic and neurological health, regulating appetite, and preventing colon cancer (51–56). Butyrate also serves as an energy source for colon cells (57).

The *in silico* predicted increase in SCFA production can be attributed to the high content of dietary fiber and phytochemicals in these foods, as suggested by other microbiome investigations *in vitro*, in animals, and clinical trials. For instance, fecal microbes of adults highly fermented pumpkin skin *in vitro*, resulting in increased production of propionate and total SCFAs (58), while adding pumpkin polyphenols into the high-fat diet of type 2 diabetic rats increased the colonic concentration of butyrate and total SCFAs in digesta (59).

Soybean, which contains insoluble dietary fiber, increased the fecal content of acetate, propionate, and butyrate in mice on a high-fat diet (60), while sweet potato, which contains non-starch polysaccharides, increased the fecal content of acetate, propionate, and isobutyrate in diarrheic mice (61). Fecal fermentations of sweet potatoes using inoculum from healthy adults increased the production of acetate and total SCFAs due to the presence of fibers (62, 63), and also increased acetate production, likely due to anthocyanins found in the purple variety (64).

Berries contain bioactive polyphenols and flavonoids, which influence the composition and function of colonic microbes. For example, blackcurrant anthocyanins decreased the ratio of Bacillota/Bacteroidota phyla in murine models (65) and increased caecal and serum concentrations of propionate and butyrate (66, 67). The fecal fermentation of different raspberry compounds, including phenolic extract and total dietary fiber, showed that SCFA production was mainly driven by polyphenol content and to a lesser extent by fiber content (68).

When combined with breastmilk, couscous resulted in the greatest increase in butyrate production, although decreased total SCFA flux. This observation may be justified by the presence of dietary fibers in durum wheat, in particular arabinoxylan (69). Interventions on adults consuming wheat arabinoxylan reported increased fecal butyrate concentration (70, 71). Similarly, the fecal fermentation of wheat cereal using inoculum from six healthy weaning infants increased the production of butyrate, although the extent of the increase varied among individuals (29).

Couscous, pumpkin, strawberries, and split peas also reduced total BCFA flux when combined with breastmilk. BCFAs, like isobutyrate and isovalerate, are produced in the colon through microbial fermentation of non-absorbed amino acids. Less is known about their influence on host health but evidence suggests a link with metabolic functions (72). BCFAs are biomarkers for protein fermentation in the distal colon (73), which can generate potential deleterious metabolites, such as ammonia and phenols (74). Colonic microbial production of BCFAs was negatively correlated with dietary insoluble fiber intake (75), which could partially explain the decreased BCFAs production observed *in silico* when adding the above fiber-rich foods to breastmilk.

For instance, an intervention with yellow pea fiber decreased the fecal concentration of the BCFA isovalerate in overweight adults (76). Nevertheless, an *in vitro* fecal fermentation of 22 plant sources of fiber, including whole cereals, seeds, and pulses, using inoculum from healthy adults, found no changes in the production of BCFAs (77). In this *in* silico study, other fiber-rich foods like sweet potato, black beans, and chickpeas also did not change the flux of BCFAs when combined with breastmilk, suggesting that other dietary compounds affect BCFA production. Indeed, a recent murine model study demonstrated that the protein source is a key factor affecting the fecal BCFA content (78).

On the other hand, when strawberries and shrimp were individually combined with breastmilk, they, respectively, showed the greatest decrease in flux of total SCFAs and the greatest increase in total BCFA flux *in silico*. These observations suggest a potential deleterious alteration in the function of colonic microbes. Evidence demonstrated that reduced SCFA fecal concentration in infancy is associated with an increased risk of diseases and allergies (16, 79, 80) while increased content of BCFAs in infants’ feces was linked to weight gain (81). These imbalances in organic acid production by the colonic microbiota during infancy may affect later life. In adults, decreased fecal concentration of SCFAs has been linked with colonic dysbiosis and diseases, such as encephalitis, Parkinson, and type 2 diabetes (46, 82, 83).

Currently, there is a lack of studies evaluating how the consumption of shrimp affects colonic microbes, while evidence found for strawberries contrasted with the *in silico* observations. Strawberry interventions did not change the fecal SCFA content in healthy adults (84) but increased the caecal production of acetate, propionate, and butyrate in mice with colitis (85).

Importantly, combining foods with other foods and with breastmilk shifted their influence on colonic microbes. The combination of pumpkin-breastmilk promoted the highest flux of total SCFAs but, when pumpkin was combined with other foods and breastmilk, resulted in little or no change in total SCFA fluxes. Among the multiple food-breastmilk combinations, blackcurrant stood out for promoting the greatest increases in SCFA production. This observation suggests that the interaction between dietary compounds composing a meal has a stronger influence on the metabolism of colonic microbes than individual foods. As foods are rarely consumed individually, dietary patterns rather than individual foods are more likely to promote alterations in colonic microbes that may affect host health (86, 87).

A recent *in vitro* study evaluated how 32 foods are fermented by the fecal microbiota of New Zealand infants at weaning age. After 24 h of fermentation, blackcurrant, pumpkin, and sweet potato increased total SCFA production, which is consistent with the *in silico* observations reported here (28). Similarly, pumpkin and blackcurrant increased the production of acetate after 24 h of fermentation using fecal inoculum from New Zealand weaning infants (27). However, none of these *in vitro* studies combined foods with human milk or with other foods and human milk, providing limited information about how foods may influence the colonic microbiota of infants when added to their pre-existing dietary patterns.

*In vivo* approaches can better evaluate the relationship between dietary patterns and the colonic microbiota, measuring eventual host health outcomes. For example, a series of studies investigated the colonic microbes of malnourished infants using animal models to identify food combinations that promoted the growth of bacterial taxa associated with healthy colonic microbial development during weaning (30, 88, 89). Observational trials evaluating dietary patterns in weaning infants associated increased dietary diversity with increased microbial diversity and consequently the stabilization of the colonic microbiome (90), and with increased SCFA production (3), highlighting the importance of introducing infants to meals composed of diverse foods.

Nevertheless, clinical trials evaluating the relationship between diet and colonic microbiome are resource- and time-consuming, also facing other limitations, such as ethical concerns, low patient recruitment and adherence to the intervention, and poor accuracy of food questionnaires. In this scenario, modeling is a useful complementary tool to evaluate conditions that cannot be investigated with traditional *in vitro* and *in vivo* methods due to technical or logistical limitations.

However, this *in silico* approach has limitations. To simulate the impact of foods on the colonic microbiota of weaning infants, average fecal microbiota data were used rather than data at the individual level. Repeating the simulations with the same dietary conditions produced similar outcomes. Consequently, it was impossible to conduct statistical analyses to assess potential differences in the effect of various food-breastmilk combinations on microbial growth and metabolite production. To reduce computational demands, a threshold of 1% of relative abundance was used to select the microbial genera included in the simulations. This resulted in a small-scale microbial community that excluded genera usually found in low abundance in the colon of weaning infants but that have key metabolic functions, such as the butyrate producers *Clostridium* and *Faecalibacterium*.

The design of food-breastmilk combinations was limited by the accuracy and availability of data in food composition databases, which predominantly focus on Western-type foods, lacking information on the diversity of food varieties and cooking/preparation methods (for a review on the limitations of food composition databases, see reference (91)). This study prioritized foods produced or available in New Zealand. However, the Virtual Metabolic Human database (92) is based on the USDA National Nutrient Database for Standard Reference Release 28 (93) and does not cover all foods consumed by weaning infants in New Zealand. For example, traditional indigenous foods like kūmara (sweet potato variety) are not covered in the database and had to be replaced by the most similar food available in the database. Furthermore, data available in the VMH database do not account for individual variability in breastmilk composition.

Our study did not consider key factors influencing the composition of the colonic microbiota in infants during the first months of life, such as the mode of delivery, breastfeeding versus infant formula feeding, and the mother’s diet (94, 95). Since our simulations focused on the weaning period (typically between 5 and 12 months of age), we assumed that these early-life factors would have a lesser impact on the infant microbiome compared to dietary changes introduced during weaning (consumption of solids and less intake of breastmilk or infant formula). Nonetheless, metagenome-scale metabolic models create personalized simulations based on input data. This methodology is adaptable and can be applied to other infant populations, potentially accounting for varying early-life influences on the microbiome in future investigations.

Another drawback is that the accuracy of the simulations presented here is dependent on the quality of the microbial metabolic reconstructions (a mathematical representation of the biochemical reactions that a microorganism can perform), which may contain missing information (31) and greatly affect the prediction capability of genome-scale metabolic models (96). Microbial relative abundance data were obtained from 16S rRNA sequencing of fecal samples. This is the most common method to assess colonic microbes of weaning infants but 16S rRNA sequencing provides resolution suitable only to the genus rank (97), while the use of feces does not accurately represent the ratios of microbial communities found in the colonic mucosa and in the proximal colon (98). Relative abundances are not independent by nature, meaning that the relative abundance of one microbe is affected by the abundance of any other microbe in the community. As a result, this may lead to a false representation of the actual microbial community.

Although the metabolic reconstructions of colonic microbes used in the simulations were validated using independent data sets (99), our simulations using MICOM were not validated experimentally (due to resource and ethical constraints) or using an external data set (due to a lack of available data). A recent study using MICOM reported agreement between fluxes of propionate and butyrate predicted *in silico* and those estimated *in vitro* (100). The package stood out among seven other static metabolic modeling tools in a qualitative and quantitative assessment (101) but did not accurately correlate growth rates predicted *in silico* with those observed *in vitro* (96). Furthermore, numerical instabilities are common in MICOM when solving quadratic programming problems in large community models (32). Near zero growth rates were observed in our simulations for the less abundant genera, indicating instabilities that are likely to alter predicted fluxes of SCFAs and BCFAs.

The lack of research evaluating the effect of food-breastmilk combinations on the colonic microbiome of weaning infants and the differences between predicted *in silico* outcomes (microbial growth rates and fluxes of metabolites) and experimental outcomes (microbial relative abundance and concentration of metabolites) limits even a qualitative comparison between *in silico* observations reported here and published *in vitro* or *in vivo* results.

To calculate the fluxes of microbial metabolites through flux balance analysis, a mass steady state was assumed, implying that there is no accumulation of substrates in the intracellular space (see review (102)). However, this assumption strongly differs from *in vitro* static conditions, where resources are depleted, and microbial products accumulate over time. Due to this assumption, parameters used in the simulation, such as substrate fluxes and microbial growth rates, are fixed and thus may not represent the rapid changes of colonic microbes in response to diet (4, 103). Dynamic flux balance analysis may be a promising alternative to better represent *in vivo* conditions (104). However, this strategy is computationally intensive when dealing with complex microbial communities (105), hence limiting its use to screen the effect of a broad range of food combinations on colonic microbes.

Finally, *in silico* predictions may diverge from the behavior of colonic microbes observed *in vitro* or *in vivo*. Both unabsorbed carbohydrates and amino acids contribute to colonic microbial production of SCFAs, but colonic microbes preferentially ferment carbohydrates (106, 107). Acetate is produced in larger quantities by most colonic commensals, while propionate and butyrate are produced in lesser amounts by only a few genera normally through cross-feeding interactions (108, 109). Reduced dietary carbohydrate intake, even if replaced with protein, ultimately decreases the production of butyrate (110).

Thus, one could expect that fiber-rich plant foods would increase the *in silico* production of SCFAs when combined with breastmilk. However, that was not the case for all food combinations here evaluated. This result may be justified by the highly individualized and varied response of colonic microbes to dietary compounds (111), particularly dietary fibers like resistant starch (112, 113), but also impacted by the limitations cited above. Therefore, further experimental investigation is essential to validate the foods and food combinations identified in this study. Nevertheless, data generated by this research provide a direction for future food-microbiome investigations and show the potential of modeling approaches to complement *in vitro* and *in vivo* techniques.

Currently, there is a lack of knowledge about how solid foods affect the colonic microbiome of weaning infants. This study evaluated for the first time how food-breastmilk combinations affect the colonic microbial production of SCFAs and BCFAs using a metagenome-scale metabolic modeling approach. By quickly and inexpensively generating insights *in silico*, this study helps the design of future research *in vitro* and *in vivo*, contributing to fill a crucial knowledge gap in infant nutrition. Furthermore, our *in silico* observations suggest that the interaction of foods composing a meal has a key influence on the colonic microbial production of SCFAs and BCFAs. This encourages future microbiome investigations to focus on the combined effect of foods on colonic microbes instead of focusing on the effect of individual food items.

## MATERIALS AND METHODS

### Software

Simulations were performed in Python (version 3.9) using the package MICOM(32) (version 0.32.5) and the integrated development environment Spyder (version 5). The solver CPLEX Optimisation Studio (IBM ILOG, version 22.1) was employed under an academic license.

### Modeling workflow

Simulations used the relative abundance of the genera composing the fecal microbiota of weaning infants, metabolic reconstructions for each taxon (a list of the biochemical reactions performed by a microorganism), and the fluxes of dietary compounds of food-breastmilk combinations (a list of nutrients composing a diet, in which their respective quantities are expressed in units of time) as inputs. Outputs were the predicted individual microbial growth rates and the fluxes of metabolites produced by the microbiota, particularly SCFAs and BCFAs.

The average relative abundance of the fecal microbiota of 14 New Zealand infants aged between 5 and 12 months, obtained from a previous *in vitro* study (28), was used to represent the colonic microbiota of weaning infants (while minimizing variations in microbiota composition between individuals). Raw 16S rRNA sequencing data (Illumina MiSeq, 2 × 250 bp paired-end reads) for the fermentation control (water, at t = 0 h) were imported from NCBI archives (BioProject PRJNA669972) using the plugin q2-fondue (114). A total of 241,611 paired-end demultiplexed reads (~80,000 sequences/sample) were imported as “.qza” artefact in QIIME2 (115) (version 2023.2), resulting in a quality score of approximately 37. DADA2 (116) was used to denoise and filter the reads, which were trimmed at 250 bp. Taxonomy assignment was carried out using the q2-feature-classifier plugin and the Greengenes2 database (117), in which amplicon sequence variants were collapsed at the genus level (see Table S5).

To reduce the numerical instability (a failure in optimally solving a numerical problem) and the processing time of the simulations, taxa were filtered to include only genera with at least 1% relative abundance. As proposed by MICOM’s authors (32), low-abundance taxa can be discarded as they are unlikely to affect the overall production of microbial metabolites but increase the computational cost of the simulations. Eleven genera remained, accounting for 95% of the relative abundance of the microbial community.

The Assembly of Gut Organisms through Reconstruction and Analysis version 2 (AGORA2) (99) metabolic reconstructions were employed in the simulations. AGORA2 reconstructions were available for 10 of these genera, representing 94% of the relative abundance used as input in the simulations. The genus rank was chosen because it suited both the resolution of 16S rRNA sequencing (97) and pan models of AGORA2 metabolic reconstructions (which are available on the MICOM’s GitHub page). Pan models of the AGORA2 metabolic reconstructions at the genus rank were built by pooling individual metabolic reconstructions for strains of colonic microbes into higher taxonomic ranks.

Fluxes of dietary compounds were generated according to a pre-defined workflow, which is available on the MICOM GitHub page. Foods included in the simulations were identified through a literature survey. Those corresponded to food items that are commonly introduced to infants at weaning (6 to 12 months old) in New Zealand (38, 40). To account for cultural variations in dietary habits, complementary foods introduced to infants in other geographical locations such as North America (37) and Europe (36) were also included. In alignment with current nutrition recommendations (39, 41), included food items were from various food groups, such as vegetables, fruits, nuts, legumes, meats, cereals, and dairy products, while food groups not recommended to infants, such as sweets and fast-food products, were excluded.

To represent how solid foods are consumed by weaning infants, identified food items were combined, individually or in pairs, with mature breastmilk using the “Design a diet” function of the Virtual Metabolic Human database (92) (VMH) (see Table S6 for the list of foods). Food varieties are prioritized for New Zealand production or local availability. When New Zealand varieties were not included in the VMH database (which is based on the USDA National Nutrient Database), they were replaced with similar available varieties.

Previous evidence estimated that at 6 months of age, the average weight of infants is 7.6 kg (118) and their energy requirement is 85 kcal/kg/day (119), of which 85% is supplied by breastmilk (119). Therefore, it was assumed that diets for 6-month-old infants would consist of 85% of breastmilk and 15% of complementary foods, totaling 608 kcal/day. In total, dietary fluxes of 155 food-breastmilk combinations and two controls (infant formula only and breastmilk only) were imported from the VMH database and processed in their original unit, mmol/day, as this reduced the numerical instability of the simulations.

Host-secreted mucin cores and the bile acids glycocholic acid and taurocholic acid were then added to the dietary fluxes (values of 1 mmol/h) to better represent the substrates available for microbes in the human colon. The next step of the MICOM workflow used the human metabolic network Recon3D (120) resource to identify the dietary compounds that are absorbed in the small intestine. The flux of these compounds was then multiplied by a factor of 0.2 to account for their intestinal absorption. Finally, AGORA2 (99) metabolic reconstructions were used to identify and add the missing nutrients necessary to allow growth rates of at least 0.01 /h for all microbial species of colonic microbes comprised by these metabolic reconstructions. This step is necessary because the dietary fluxes provided by food databases often lack essential cofactors for microbial growth, such as vitamins and minerals. After supplementing the fluxes with the minimal additional substrates (which are added in mmol/h), the final fluxes were in mmol/h.

### Modelling assumptions

This modeling approach was based on flux balance analysis under a mass steady-state assumption, meaning that there is no accumulation of substances inside the microbial cells (102). A constrained linear programming problem (a mathematical problem) for the fluxes (*v*) was created using a stoichiometric matrix (S), which is a matrix representing all biochemical reactions (as columns) and involved metabolites (as rows) performed by the microbial community. The objective of the flux balance analysis was to maximize biomass reaction (*v*_bm_) such that there is no accumulation of substrates in the system (S*v = 0).

The steady-state assumption represents the exponential phase of bacterial growth, in which growth rates can be assumed constant, and the linear problem describing fluxes of metabolites can be solved by a programming solver. The community growth rate (µ_c_) is determined by the sum of growth rates of individual microbes (µ_i_) weighted by their relative abundances (a_i_), as described by the following equation:


$$\mu_{c}=\sum_{i}a_{i}\mu_{i}$$

MICOM also distributes growth across all members of the microbial community by limiting the maximal community growth with a cooperative trade-off (a value between 0 and 1). The optimal trade-off between maximal microbial community growth and maximal individual microbial growth was determined for each food-breastmilk combination. This allows most microbes present in the community to grow, rather than only the dominant members, thus better representing *in vivo* conditions in which multiple microbes are found in different abundances in the human colon (11). The cooperative trade-off (α) was calculated in two steps, initially determining the maximal community growth rate (µ_c_^max^) and then calculating the optimal individual growth rates (µ_i_^opt^), which are positively correlated with the relative abundance (a) of individual taxa (32):


$$\mu_{i}^{opt}=\frac{\alpha \;\mu_{c}^{max}}{a^{T}\;a}\;a$$

MICOM assumes that the relative abundance of microbes present in a sample corresponds to their relative biomass, in which the biomass reaction is normalized to produce 1 g, predicting the production of microbial metabolites in mmoles/g.h (of microbial biomass in dry weight) (for further description see reference (32)). The total flux of a given microbial metabolite (*v*_tot_^m^) is calculated as the sum of the fluxes of this metabolite produced by individual microbes (*v*_i_^m^) weighted by their relative abundances (a_i_), as described by the equation below:


$$v_{tot}^{m}=\sum_{i}a_{i}\;v_{i}^{m}$$

### Criteria for selecting food-breastmilk combinations

To identify food and food combinations having the strongest influence on the colonic microbiome of New Zealand weaning infants, this study focused on the production of microbial metabolites (microbial function) rather than changes in microbial composition. This is due to the potential of functional analyses to be more informative about how diets shape colonic microbes. Indeed, evidence linked variations in the genetic content of colonic commensals between individuals with their ability to metabolize dietary compounds and produce bioactive metabolites (121, 122).

Predicted microbial fluxes of the major SCFAs (acetate, propionate, and butyrate) and BCFAs (isovalerate and isobutyrate) were chosen due to the importance of these organic acids on host physiology and because their concentration is known to be influenced by host dietary patterns during weaning (49, 123). As criteria of choice, selected foods and food combinations were representative of different model outputs, corresponding to candidates that, when combined with breastmilk, resulted in the greatest increase, decrease, or did not change the fluxes of total SCFAs and BCFAs, in comparison to breastmilk alone. Foods commonly consumed by weaning infants in New Zealand and produced in the country were prioritized.

## SUPPLEMENTAL MATERIAL

Supplemental tables msystems.00577-24-s0001.pdf Tables S1 to S6.

ASM does not own the copyrights to Supplemental Material that may be linked to, or accessed through, an article. The authors have granted ASM a non-exclusive, world-wide license to publish the Supplemental Material files. Please contact the corresponding author directly for reuse.
