# Supplementary material for: Food-breastmilk combinations alter the colonic microbiome of weaning infants: an in silico study
Source: mSystems. 2024 Aug 27;9(9):e00577-24. doi: 10.1128/msystems.00577-24 (PMC11406890; doi:10.1128/msystems.00577-24)
Supplement: Supplemental tables — Tables S1 to S6. [file msystems.00577-24-s0001.pdf]

SUPPLEMENTARY TABLE 1 Predicted fluxes of SCFAs and BCFAs according to different food-breastmilk combinations.

Food-breastmilk combination	Fluxes of organic acids (mmol/gDW.h)						
	Acetate	Propionate	Butyrate	SCFAs	Isobutyrate	Isovalerate	BCFAs
Control Breastmilk	264.1	65.5	29.3	358.9	9.2	6.4	15.6
Control Infant formula	256.0	69.0	30.9	355.9	9.1	6.3	15.4
Broccoli	243.1	77.9	28.2	349.2	9.1	5.9	15.1
Brussel	272.1	61.7	33.0	366.7	8.9	6.4	15.4
Cabbage	265.6	68.9	29.9	364.4	9.1	6.5	15.6
Carrot	264.3	37.0	8.7	310.0	8.3	5.5	13.8
Cauliflower	254.4	74.3	29.0	357.6	8.9	6.1	15.0
Celery	257.3	66.1	24.7	348.1	6.6	5.9	12.6
Cucumber	242.3	43.9	45.3	331.5	7.7	5.4	13.0
Eggplant	258.4	69.8	27.6	355.8	9.2	6.4	15.6
Green beans	243.4	79.2	28.5	351.1	9.2	6.2	15.4
Green capsicum	254.4	55.6	28.7	338.7	8.6	5.7	14.3
Lettuce	254.2	67.5	27.1	348.8	9.3	6.5	15.8
Mushroom	256.0	70.3	27.1	353.4	9.3	6.6	15.8
Onion	255.1	69.5	28.9	353.6	9.4	6.3	15.7
Pak choi	248.7	75.7	28.2	352.6	9.5	6.5	15.9
Potato	288.9	54.7	24.3	367.9	8.5	5.9	14.4
Pumpkin	257.0	108.1	35.8	400.8	6.1	3.2	9.3
Sweetcorn	288.3	54.9	22.5	365.8	7.6	5.9	13.5
Spinach	177.1	120.6	18.9	316.6	5.3	6.9	12.2
Squash	268.2	64.9	26.9	360.1	8.1	6.6	14.7
Sweet potato	281.9	60.4	27.3	369.6	9.2	6.3	15.5
Tomato	261.9	66.8	27.7	356.4	9.4	6.5	15.9
Yam	264.8	73.7	32.8	371.3	9.8	6.6	16.4
Zucchini	255.9	61.2	24.2	341.3	8.2	5.9	14.1
Apple	266.5	65.2	28.5	360.2	8.9	6.3	15.2
Banana	254.0	71.4	28.5	353.9	9.1	6.4	15.5
Blackcurrant	299.3	52.2	29.6	381.1	8.8	6.7	15.6
Blueberries	252.1	70.8	30.0	352.9	9.0	6.1	15.0
Cherry	257.9	70.2	29.2	357.3	9.3	6.4	15.7

Feijoa	264.2	60.2	30.4	354.7	9.2	6.4	15.6
Gold kiwifruit	292.0	37.2	25.1	354.3	8.6	6.7	15.3
Grape	261.8	68.4	28.1	358.3	8.8	6.3	15.0
Grapefruit	240.4	85.3	27.9	353.6	8.7	6.0	14.7
Green kiwifruit	263.4	63.3	27.4	354.2	8.8	6.4	15.2
Mandarin	238.6	66.0	24.8	329.4	8.6	6.0	14.6
Mango	259.8	66.7	29.0	355.5	9.7	6.5	16.1
Melon	261.5	64.2	28.6	354.3	9.3	6.3	15.6
Nectarine	237.4	79.9	30.2	347.5	9.0	6.2	15.3
Orange	244.9	69.1	27.5	341.5	9.2	6.4	15.5
Peach	259.0	70.9	30.1	360.1	9.1	6.5	15.6
Pear	255.1	19.4	15.6	290.0	5.7	8.2	13.9
Pineapple	273.1	60.0	27.4	360.4	9.5	6.3	15.7
Plum	250.4	49.3	20.2	319.9	8.3	6.7	15.0
Raspberries	282.7	69.5	29.6	381.8	9.3	6.2	15.5
Strawberries	105.1	24.4	10.9	140.3	5.8	4.5	10.3
Barley	250.8	74.0	27.7	352.5	8.7	6.4	15.1
Barley cereal	282.6	61.4	25.1	369.2	8.8	5.9	14.8
Couscous	215.8	23.9	60.1	299.8	4.1	1.9	6.0
Noodles	252.8	70.7	29.4	352.9	9.3	6.4	15.6
Oat cereal	253.1	74.5	27.0	354.5	9.0	6.4	15.5
Pasta	239.6	78.8	29.8	348.3	9.3	6.4	15.7
Rice	233.7	56.5	16.5	306.7	7.5	5.4	13.0
Rice cereal	263.6	74.4	35.9	373.9	9.9	6.7	16.6
Tapioca pudding	263.2	69.0	30.1	362.4	9.1	6.3	15.4
White bread	250.8	70.4	30.5	351.6	9.1	6.3	15.4
Wholegrain bread	171.6	36.0	17.9	225.4	7.7	6.4	14.1
Cottage cheese	259.6	69.6	31.5	360.7	9.6	6.4	16.0
Eggs	251.3	73.8	29.6	354.7	9.2	6.3	15.5
Mozzarella cheese	265.8	69.3	30.5	365.6	9.3	6.3	15.6
Soymilk	261.3	70.1	27.3	358.6	8.6	6.2	14.8
Tofu	257.4	70.1	29.2	356.7	9.3	6.5	15.8
Whole milk	264.9	57.1	27.1	349.1	8.6	6.4	15.0
Yoghurt	248.1	53.9	24.8	326.7	8.0	5.5	13.5

Beef	261.3	65.9	30.0	357.2	9.0	6.3	15.3
Chicken	264.3	71.6	31.8	367.6	9.8	6.7	16.4
Codfish	248.3	68.4	23.0	339.7	8.5	6.0	14.5
Lamb	262.6	66.5	35.8	364.8	9.3	6.5	15.8
Mackerel	274.5	55.1	27.8	357.4	9.0	6.3	15.4
Mussels	265.6	70.0	29.2	364.8	9.0	6.4	15.4
Pork	261.1	70.3	28.8	360.2	9.3	6.4	15.6
Salmon	251.3	73.5	31.8	356.7	9.2	6.3	15.5
Shrimp	264.3	38.1	23.1	325.6	11.7	7.5	19.2
Tuna	258.9	69.8	28.7	357.4	9.1	6.3	15.4
Turkey	267.2	64.0	28.3	359.4	9.2	6.2	15.3
Almond	223.4	73.1	29.8	326.3	9.2	6.1	15.3
Black beans	259.2	70.7	28.1	358.0	9.2	6.4	15.6
Cashew	257.7	62.9	28.5	349.1	8.5	6.4	14.9
Chia	260.7	67.0	28.7	356.4	8.9	6.4	15.3
Chickpea	258.1	71.6	29.7	359.4	9.3	6.4	15.7
Green peas	256.7	67.8	27.0	351.5	8.9	6.2	15.0
Hazelnut	165.4	27.9	21.8	215.0	7.0	7.0	14.1
Lentils	252.5	61.9	26.6	341.0	8.7	5.9	14.6
Peanut	294.1	46.0	28.0	368.1	8.3	6.1	14.4
Pecans	221.1	33.4	21.6	276.1	5.2	4.2	9.4
Pumpkin seed	244.3	71.2	28.2	343.7	9.0	6.2	15.2
Red beans	267.5	65.8	27.5	360.8	9.3	6.4	15.7
Soybean	277.7	67.5	28.1	373.3	9.0	6.5	15.4
Split peas	297.9	33.7	33.7	365.3	5.2	6.2	11.4
Sunflower seed	237.4	54.7	21.3	313.4	8.4	7.7	16.1
White beans	265.7	67.5	30.9	364.1	9.2	6.5	15.7

Values are coloured according to intensity for each organic acid, with the highest values in green and the lowest values in red.

SUPPLEMENTARY TABLE 2 Predicted microbial growth rates according to different food-breastmilk combinations.

Food-breastmilk combination	Growth rate (1/h)									
	Bacillus	*Bacteroides*	*Bifidobacterium*	*Collinsella*	*Lacticaseibacillus*	*Lactobacillus*	*Limosilactobacillus*	*Prevotella*	*Streptococcus*	*Veillonella*
Control Breastmilk	0.064	0.068	0.103	0.009		0.005		0.019		
Control Infant formula	0.045	0.065	0.080	0.008	0.006	0.004	0.003	0.013	0.003	0.028
Broccoli	0.057	0.070	0.091	0.008	0.002	0.004	0.003	0.019		
Brussel	0.051	0.069	0.087	0.009	0.007	0.004	0.003	0.015	0.001<	0.009
Cabbage	0.058	0.070	0.091	0.009	0.001	0.005	0.003	0.018		
Carrot	0.050	0.067	0.085	0.009	0.009	0.004	0.003	0.015	0.001	0.015
Cauliflower	0.049	0.068	0.085	0.008	0.006	0.004	0.003	0.015	0.001	0.015
Celery	0.059	0.068	0.091	0.008	0.002	0.005	0.003	0.019		
Cucumber	0.057	0.068	0.092	0.008	0.005	0.005	0.003	0.018		
Eggplant	0.050	0.068	0.086	0.008	0.006	0.004	0.003	0.015	0.001	0.014
Green beans	0.054	0.071	0.091	0.009	0.004	0.004	0.003	0.017	0.001<	0.001<
Green capsicum	0.052	0.068	0.087	0.008	0.011	0.004	0.003	0.016		0.010
Lettuce	0.050	0.068	0.086	0.009	0.006	0.004	0.003	0.015	0.001	0.013
Mushroom	0.054	0.071	0.090	0.009	0.004	0.004	0.003	0.016		0.003
Onion	0.055	0.071	0.091	0.009	0.001<	0.004	0.003	0.017		0.001<
Pak choi	0.073	0.063	0.103	0.006		0.005	0.001	0.022		
Potato	0.067	0.066	0.103	0.008		0.005	0.002	0.020		
Pumpkin	0.053	0.070	0.090	0.009	0.002	0.004	0.003	0.016		0.004
Sweetcorn	0.057	0.069	0.091	0.008	0.003	0.005	0.003	0.020		
Spinach	0.055	0.071	0.091	0.009	0.003	0.004	0.003	0.016		
Squash	0.062	0.080	0.103	0.010	0.002	0.005	0.003	0.018	0.001<	0.001<
Sweet potato	0.058	0.069	0.091	0.008	0.001	0.005	0.003	0.018		
Tomato	0.051	0.068	0.086	0.009	0.010	0.004	0.003	0.016	0.001	0.011
Yam	0.060	0.068	0.091	0.009		0.005	0.002	0.018		
Zucchini	0.030	0.043	0.053	0.006	0.008	0.002	0.002	0.009	0.002	0.038
Apple	0.060	0.067	0.091	0.008		0.004	0.002	0.020		
Banana	0.050	0.068	0.086	0.008	0.005	0.004	0.003	0.015	0.001	0.014
Blackcurrant	0.063	0.068	0.103	0.009		0.005		0.019		
Blueberries	0.046	0.065	0.081	0.008	0.007	0.004	0.003	0.013	0.003	0.027
Cherry	0.057	0.078	0.098	0.010	0.003	0.004	0.003	0.016	0.001	0.004
Feijoa	0.050	0.068	0.086	0.008	0.004	0.004	0.003	0.015	0.001	0.012
Gold kiwifruit	0.050	0.067	0.085	0.008	0.009	0.004	0.003	0.015	0.001	0.014
Grape	0.084	0.066	0.103	0.003		0.005	0.001<	0.027		
Grapefruit	0.059	0.068	0.091	0.008		0.005	0.003	0.019		
Green kiwifruit	0.050	0.068	0.086	0.008	0.004	0.004	0.003	0.014	0.001	0.014
Mandarin	0.052	0.069	0.088	0.009	0.007	0.004	0.003	0.016	0.001<	0.007

Mango	0.052	0.069	0.088	0.009	0.006	0.004	0.003	0.015	0.001	0.009
Melon	0.049	0.068	0.085	0.008	0.006	0.004	0.003	0.015	0.001	0.015
Nectarine	0.057	0.078	0.098	0.009	0.005	0.004	0.003	0.016	0.001	0.001
Orange	0.072	0.064	0.102	0.007		0.005		0.023		
Peach	0.050	0.068	0.085	0.009	0.009	0.004	0.003	0.015	0.001	0.013
Pear	0.052	0.068	0.087	0.009	0.003	0.004	0.003	0.016	0.001<	0.010
Pineapple	0.051	0.069	0.088	0.009	0.008	0.004	0.003	0.016	0.001	0.008
Plum	0.044	0.064	0.079	0.008	0.007	0.003	0.003	0.012	0.003	0.028
Raspberries	0.057	0.069	0.091	0.008	0.004	0.005	0.003	0.019		
Strawberries	0.057	0.070	0.091	0.008	0.002	0.005	0.003	0.018		
Barley	0.051	0.069	0.087	0.009	0.006	0.004	0.003	0.015	0.001	0.009
Barley cereal	0.065	0.067	0.103	0.009		0.005	0.003	0.019		
Couscous	0.049	0.068	0.085	0.009	0.007	0.004	0.003	0.014	0.001	0.015
Noodles	0.049	0.067	0.085	0.008	0.006	0.004	0.003	0.014	0.002	0.016
Oat cereal	0.037	0.054	0.067	0.007	0.009	0.003	0.003	0.011	0.003	0.032
Pasta	0.064	0.071	0.103	0.009		0.005		0.019		
Rice	0.037	0.050	0.062	0.006	0.006	0.003	0.002	0.011	0.001	0.015
Rice cereal	0.049	0.067	0.085	0.008	0.005	0.004	0.003	0.014	0.001	0.017
Tapioca pudding	0.052	0.070	0.088	0.009	0.004	0.004	0.003	0.016	0.001<	0.007
White bread	0.050	0.068	0.086	0.009	0.006	0.004	0.003	0.014	0.001	0.014
Wholegrain bread	0.059	0.068	0.091	0.008		0.005	0.002	0.018		
Cottage cheese	0.050	0.068	0.085	0.009	0.007	0.004	0.003	0.015	0.001	0.014
Eggs	0.053	0.071	0.090	0.009	0.003	0.004	0.003	0.016	0.001<	0.003
Mozzarella cheese	0.068	0.063	0.091	0.006		0.005	0.001	0.022		
Soymilk	0.050	0.068	0.086	0.008	0.007	0.004	0.003	0.015	0.001	0.014
Tofu	0.038	0.055	0.068	0.007	0.009	0.003	0.003	0.011	0.003	0.030
Whole milk	0.049	0.068	0.085	0.008	0.007	0.004	0.003	0.014	0.001	0.015
Yoghurt	0.051	0.068	0.086	0.009	0.007	0.004	0.003	0.015	0.001	0.012
Beef	0.051	0.069	0.087	0.008	0.003	0.004	0.003	0.014	0.001	0.012
Chicken	0.049	0.068	0.085	0.008	0.007	0.004	0.003	0.014	0.001	0.015
Codfish	0.054	0.069	0.089	0.009	0.006	0.004	0.003	0.017		0.005
Lamb	0.042	0.057	0.072	0.007	0.008	0.003	0.003	0.012	0.002	0.019
Mackerel	0.054	0.071	0.090	0.009	0.006	0.004	0.003	0.016		0.001
Mussels	0.067	0.067	0.103	0.008		0.005		0.020		
Pork	0.086	0.065	0.102	0.004	0.001<	0.005	0.001	0.028		
Salmon	0.051	0.069	0.087	0.009	0.006	0.004	0.003	0.015	0.001	0.011
Shrimp	0.050	0.068	0.086	0.008	0.007	0.004	0.003	0.014	0.001	0.013
Tuna	0.054	0.071	0.090	0.009	0.004	0.004	0.003	0.016		0.002
Turkey	0.051	0.069	0.087	0.008	0.005	0.004	0.003	0.015	0.001	0.011
Almond	0.066	0.064	0.092	0.006		0.005	0.001	0.021		
Black beans	0.064	0.069	0.103			0.005		0.019		

Cashew	0.042	0.057	0.072	0.007	0.007	0.003	0.003	0.013	0.002	0.018
Chia	0.052	0.070	0.088	0.009	0.007	0.004	0.003	0.016	0.001<	0.007
Chickpea	0.053	0.070	0.089	0.009	0.004	0.004	0.003	0.016	0.001<	0.005
Green beans	0.054	0.071	0.091	0.009	0.004	0.004	0.003	0.017	0.001<	0.001<
Hazelnut	0.063	0.066	0.091	0.007		0.005	0.002	0.021		
Lentils	0.050	0.069	0.087	0.009	0.004	0.004	0.003	0.015	0.001	0.011
Peanut	0.062	0.080	0.103	0.009	0.001<	0.004	0.003	0.018		
Pecans	0.065	0.065	0.091	0.006		0.005	0.001	0.022		
Pumpkin seed	0.062	0.071	0.103		0.001	0.004	0.003	0.018		
Red beans	0.050	0.068	0.086	0.009	0.006	0.004	0.003	0.014	0.001	0.012
Soybean	0.066	0.066	0.103	0.008		0.005		0.020		
Split peas	0.055	0.071	0.091	0.008	0.004	0.005	0.003	0.018		
Sunflower seed	0.025	0.036	0.044	0.005	0.007	0.002	0.002	0.007	0.003	0.032
White beans	0.050	0.068	0.085	0.009	0.006	0.004	0.003	0.015	0.001	0.014

Values are coloured according to intensity for each genera, with the highest values in green and the lowest values in red. Blank cells correspond to absent growth.

SUPPLEMENTARY TABLE 3 Predicted fluxes of SCFAs and BCFAs for multiple food-breastmilk combinations.

Multiple food-breastmilk combination	Fluxes of organic acids (mmol/gDW.h)						
	Acetate	Propionate	Butyrate	SCFAs	Isobutyrate	Isovalerate	BCFAs
Control Breastmilk	264.1	65.5	29.3	358.9	9.2	6.4	15.6
Control Infant formula	256.0	69.0	30.9	355.9	9.1	6.3	15.4
Black beans and Blackcurrant	254.5	51.0	20.6	326.1	10.6	6.5	17.1
Black beans and Chickpea	242.9	32.7	14.0	289.6	6.5	6.9	13.4
Black beans and Couscous	254.6	72.2	31.2	358.0	9.2	6.3	15.4
Black beans and Pork	246.8	71.9	28.0	346.7	9.2	6.3	15.5
Black beans and Pumpkin	115.4	17.0	149.8	282.3	1.9	0.5	2.4
Black beans and Raspberries	275.2	62.3	27.6	365.1	8.4	6.2	14.6
Black beans and Shrimp	255.5	74.4	28.7	358.6	9.5	6.4	15.9
Black beans and Soybean	239.5	67.5	33.0	340.0	9.5	6.5	16.0
Black beans and Split peas	258.5	70.8	28.2	357.5	9.3	6.4	15.6
Black beans and Strawberries	258.3	70.1	31.7	360.1	9.3	6.4	15.7
Black beans and Sweet potato	269.7	55.0	24.9	349.6	7.4	5.5	12.9
Blackcurrant and Chickpea	250.2	69.8	27.4	347.4	9.2	6.0	15.1
Blackcurrant and Couscous	261.4	60.9	29.6	351.9	9.1	6.5	15.6
Blackcurrant and Pork	263.7	31.2	52.7	347.6	5.2	3.0	8.2
Blackcurrant and Pumpkin	253.1	75.5	30.3	358.9	9.3	6.4	15.7
Blackcurrant and Raspberries	260.0	62.8	29.0	351.8	8.7	6.1	14.8
Blackcurrant and Shrimp	254.4	70.4	27.2	351.9	8.7	6.2	14.9
Blackcurrant and Soybean	311.9	61.6	27.5	401.0	8.4	6.5	14.9
Blackcurrant and Split peas	257.8	68.7	26.9	353.4	8.6	6.3	14.9
Blackcurrant and Strawberries	301.7	53.6	28.3	383.6	9.4	6.5	15.9
Blackcurrant and Sweet potato	297.1	60.4	25.2	382.7	8.3	6.0	14.3
Chickpea and Couscous	253.0	76.4	26.5	355.9	7.7	5.6	13.2
Chickpea and Pork	277.7	37.0	49.2	363.9	6.1	3.8	9.9
Chickpea and Pumpkin	257.6	74.5	31.6	363.7	9.2	6.2	15.4
Chickpea and Raspberries	255.2	69.8	30.5	355.5	9.3	6.3	15.6
Chickpea and Shrimp	260.3	70.6	30.0	360.9	9.3	6.4	15.7
Chickpea and Soybean	260.6	72.4	30.8	363.8	9.3	6.4	15.8
Chickpea and Split peas	68.9	19.2	12.5	100.6	2.5	1.3	3.8
Chickpea and Strawberries	258.3	70.2	30.0	358.6	9.5	6.4	15.9

Chickpea and Sweet potato	256.2	73.1	28.3	357.6	9.1	6.4	15.5
Couscous and Pork	277.7	54.7	15.2	347.6	6.7	4.8	11.5
Couscous and Pumpkin	257.2	71.6	30.4	359.2	9.1	6.4	15.5
Couscous and Raspberries	258.0	67.7	26.5	352.2	9.1	6.1	15.2
Couscous and Shrimp	279.5	58.9	26.9	365.3	9.0	6.3	15.3
Couscous and Soybean	261.5	70.7	31.1	363.3	9.1	6.4	15.5
Couscous and Split peas	255.8	67.9	33.8	357.6	8.8	6.6	15.3
Couscous and Strawberries	247.6	67.9	27.7	343.2	8.9	5.9	14.9
Couscous and Sweet potato	238.7	63.0	7.4	309.1	7.4	5.6	13.1
Pork and Pumpkin	258.6	71.0	32.0	361.6	9.2	6.3	15.6
Pork and Raspberries	282.0	64.1	28.0	374.1	9.0	6.3	15.2
Pork and Shrimp	259.6	67.3	30.7	357.6	9.3	6.4	15.7
Pork and Soybean	260.1	68.3	30.0	358.4	9.3	6.4	15.7
Pork and Split peas	269.1	75.7	33.0	377.9	9.8	6.5	16.3
Pork and Strawberries	290.2	34.6	25.6	350.4	7.6	6.1	13.7
Pork and Sweet potato	278.2	59.3	27.8	365.3	9.2	6.5	15.7
Pumpkin and Raspberries	260.1	77.7	30.3	368.1	9.2	6.6	15.8
Pumpkin and Shrimp	290.1	43.7	28.5	362.3	8.6	7.3	15.8
Pumpkin and Soybean	250.0	74.7	30.7	355.3	9.4	6.5	15.9
Pumpkin and Split peas	258.9	71.0	29.4	359.3	9.2	6.4	15.6
Pumpkin and Strawberries	249.3	76.2	29.3	354.8	9.2	6.4	15.6
Pumpkin and Sweet potato	270.7	61.2	28.7	360.5	8.9	6.2	15.2
Raspberries and Shrimp	268.2	55.8	24.1	348.1	8.9	6.5	15.4
Raspberries and Soybean	255.2	44.5	20.7	320.3	8.9	6.1	14.9
Raspberries and Split peas	256.7	59.2	27.2	343.0	8.4	6.0	14.4
Raspberries and Strawberries	203.8	25.1	19.6	248.4	7.3	6.6	13.9
Raspberries and Sweet potato	229.2	91.1	28.4	348.7	9.3	6.6	15.9
Shrimp and Soybean	272.0	54.8	27.9	354.7	9.1	6.8	15.9
Shrimp and Split peas	260.3	70.9	26.9	358.1	9.1	6.3	15.4
Shrimp and Strawberries	252.1	71.9	30.1	354.0	9.2	6.4	15.6
Shrimp and Sweet potato	247.0	70.5	29.2	346.7	9.2	6.3	15.5
Soybean and Split peas	272.0	72.6	27.8	372.3	8.7	6.2	14.9
Soybean and Strawberries	260.0	72.5	30.5	362.9	9.5	6.6	16.0
Soybean and Sweet potato	260.1	52.0	25.2	337.3	8.3	6.0	14.3

Split peas and Strawberries	255.3	67.9	29.3	352.5	9.2	6.3	15.4
Split peas and Sweet potato	265.0	69.4	26.4	360.8	9.0	6.4	15.4
Strawberries and Sweet potato	251.8	68.3	28.6	348.7	8.9	6.3	15.3

Values are coloured according to intensity for each organic acid, with the highest values in green and the lowest values in red.

SUPPLEMENTARY TABLE 4 Predicted microbial growth rates for multiple food-breastmilk combinations and controls.

Multiple food-breastmilk combination	Growth rate (1/h)									
	Bacillus	*Bacteroides*	*Bifidobacterium*	*Collinsella*	*Lacticaseibacillus*	*Lactobacillus*	*Limosilactobacillus*	*Prevotella*	*Streptococcus*	*Veillonella*
Control Breastmilk	0.064	0.068	0.103	0.009		0.005		0.019		
Control Infant formula	0.045	0.065	0.080	0.008	0.006	0.004	0.003	0.013	0.003	0.028
Black beans and Blackcurrant	0.015	0.022	0.027	0.003	0.004	0.001	0.001	0.004	0.002	0.018
Black beans and Chickpea	0.05	0.068	0.086	0.009	0.006	0.004	0.003	0.015	0.001	0.013
Black beans and Couscous	0.031	0.045	0.056	0.006	0.008	0.002	0.002	0.009	0.003	0.031
Black beans and Pork	0.067	0.067	0.103	0.008		0.004		0.020		
Black beans and Pumpkin	0.045	0.060	0.076	0.008	0.005	0.004	0.003	0.014	0.001	0.010
Black beans and Raspberries	0.052	0.069	0.087	0.009	0.008	0.004	0.003	0.015	0.001	0.009
Black beans and Shrimp	0.056	0.070	0.091	0.008	0.003	0.004	0.003	0.018		
Black beans and Soybean	0.072	0.063	0.103	0.007		0.005		0.022		
Black beans and Split peas	0.065	0.069	0.103			0.005	0.003	0.020		
Black beans and Strawberries	0.148	0.226	0.276	0.030	0.043	0.011	0.011	0.040	0.021	0.184
Black beans and Sweet potato	0.055	0.068	0.089	0.009	0.005	0.004	0.003	0.018		0.006
Blackcurrant and Chickpea	0.051	0.069	0.087	0.009	0.006	0.004	0.003	0.015	0.001	0.011
Blackcurrant and Couscous	0.052	0.070	0.089	0.009	0.005	0.004	0.003	0.015	0.001<	0.006
Blackcurrant and Pork	0.070	0.064	0.103	0.007		0.005	0.002	0.022		
Blackcurrant and Pumpkin	0.051	0.069	0.087	0.008	0.004	0.004	0.003	0.015	0.001	0.011
Blackcurrant and Raspberries	0.053	0.070	0.089	0.009	0.006	0.004	0.003	0.017		0.005
Blackcurrant and Shrimp	0.056	0.070	0.091	0.008	0.006	0.005	0.003	0.017		
Blackcurrant and Soybean	0.066	0.077	0.101	0.008		0.005		0.020		
Blackcurrant and Split peas	0.043	0.058	0.073	0.008	0.007	0.004	0.003	0.013	0.002	0.015
Blackcurrant and Strawberries	0.058	0.069	0.091	0.008		0.005	0.003	0.018		
Blackcurrant and Sweet potato	0.082	0.067	0.103	0.004		0.006	0.001	0.026		
Chickpea and Couscous	0.076	0.061	0.103	0.006		0.004	0.001	0.023		
Chickpea and Pork	0.049	0.068	0.085	0.008	0.007	0.004	0.003	0.015	0.002	0.014
Chickpea and Pumpkin	0.051	0.068	0.087	0.009	0.006	0.004	0.003	0.015	0.001	0.011
Chickpea and Raspberries	0.044	0.058	0.074	0.008	0.009	0.004	0.003	0.014	0.001<	0.015
Chickpea and Shrimp	0.143	0.197	0.248	0.024	0.014	0.010	0.008	0.040	0.002	0.037
Chickpea and Soybean	0.050	0.068	0.086	0.008	0.005	0.004	0.003	0.015	0.001	0.013

Chickpea and Split peas	0.020	0.031	0.035	0.004	0.006	0.002	0.001	0.006	0.002	0.024
Chickpea and Strawberries	0.214	0.297	0.372	0.036	0.019	0.015	0.012	0.059	0.004	0.065
Chickpea and Sweet potato	0.058	0.069	0.091	0.008	0.001	0.005	0.003	0.019		
Couscous and Pork	0.050	0.068	0.086	0.008	0.006	0.004	0.003	0.015	0.001	0.012
Couscous and Pumpkin	0.044	0.059	0.074	0.008	0.006	0.004	0.003	0.013	0.001	0.013
Couscous and Raspberries	0.049	0.067	0.084	0.008	0.006	0.004	0.003	0.014	0.002	0.018
Couscous and Shrimp	0.098	0.098	0.152	0.012		0.006	0.003	0.029		
Couscous and Soybean	0.063	0.070	0.103			0.005	0.003	0.018		
Couscous and Split peas	0.039	0.055	0.069	0.007	0.007	0.003	0.003	0.011	0.003	0.028
Couscous and Strawberries	0.057	0.070	0.091	0.008	0.001	0.005	0.003	0.017		
Couscous and Sweet potato	0.053	0.071	0.090	0.009	0.003	0.004	0.003	0.015	0.001<	0.004
Pork and Pumpkin	0.053	0.070	0.089	0.009	0.005	0.004	0.003	0.016		0.004
Pork and Raspberries	0.065	0.065	0.091	0.006		0.005	0.003	0.021		
Pork and Shrimp	0.033	0.047	0.059	0.006	0.009	0.003	0.002	0.010	0.003	0.023
Pork and Soybean	0.051	0.069	0.087	0.009	0.006	0.004	0.003	0.015	0.001	0.011
Pork and Split peas	0.05	0.068	0.085	0.009	0.007	0.004	0.003	0.015	0.001	0.015
Pork and Strawberries	0.057	0.07	0.091	0.008	0.003	0.005	0.003	0.018		
Pork and Sweet potato	0.055	0.071	0.091	0.009	0.004	0.004	0.003	0.017		
Pumpkin and Raspberries	0.063	0.066	0.091	0.007		0.005	0.002	0.022		
Pumpkin and Shrimp	0.032	0.045	0.057	0.006	0.009	0.002	0.002	0.009	0.003	0.029
Pumpkin and Soybean	0.053	0.070	0.089	0.009	0.006	0.004	0.003	0.016	0.001<	0.005
Pumpkin and Split peas	0.067	0.069	0.103	0.008		0.005	0.002	0.020		
Pumpkin and Strawberries	0.079	0.069	0.100	0.005		0.005		0.026		
Pumpkin and Sweet potato	0.086	0.056	0.102	0.003		0.005		0.028		
Raspberries and Shrimp	0.060	0.068	0.091	0.008		0.005	0.002	0.019		
Raspberries and Soybean	0.052	0.070	0.089	0.009	0.008	0.004	0.003	0.016	0.001<	0.006
Raspberries and Split peas	0.055	0.070	0.090	0.009	0.005	0.004	0.003	0.017		0.001
Raspberries and Strawberries	0.052	0.069	0.088	0.009	0.008	0.004	0.003	0.016	0.001<	0.008
Raspberries and Sweet potato	0.058	0.070	0.091	0.008		0.004	0.003	0.018		
Shrimp and Soybean	0.038	0.050	0.064	0.007	0.007	0.003	0.002	0.012	0.001	0.010
Shrimp and Split peas	0.050	0.068	0.085	0.009	0.007	0.004	0.003	0.015	0.001<	0.015
Shrimp and Strawberries	0.056	0.070	0.091	0.009	0.003	0.004	0.003	0.017		
Shrimp and Sweet potato	0.055	0.071	0.091	0.009	0.002	0.004	0.003	0.017		
Soybean and Split peas	0.055	0.071	0.091	0.009	0.003	0.004	0.003	0.017		
Soybean and Strawberries	0.172	0.238	0.298	0.029	0.013	0.012	0.009	0.048	0.003	0.050

Soybean and Sweet potato	0.058	0.069	0.091	0.008	0.001<	0.005	0.003	0.018		
Split peas and Strawberries	0.228	0.317	0.398	0.039	0.013	0.016	0.012	0.062	0.006	0.081
Split peas and Sweet potato	0.094	0.051	0.102	0.002		0.004	0.001<	0.030		
Strawberries and Sweet potato	0.059	0.068	0.092	0.008		0.005	0.003	0.019		

Values are coloured according to intensity for each genera, with the highest values in green and the lowest values in red. Blank cells correspond to absent growth.

SUPPLEMENTARY TABLE 5 Average relative abundance of the faecal microbiota of 14 New Zealand weaning infants used in the simulations.

Genus	Relative abundance
Bifidobacterium	0.173518
Bacteroides	0.143229
Veillonella	0.131564
Bacillus	0.092016
Lacticaseibacillus	0.028031
Prevotella	0.024361
Collinsella	0.019328
Streptococcus	0.01494
Succinispira	0.008924
Limosilactobacillus	0.007138
Lactobacillus	0.006921
Erysipelatoclostridium	0.005582
Paramuribaculum	0.003222
Clostridium	0.003161
Anaeroglobus	0.002221
Cupriavidus	0.001675
Asaccharospora	0.001577
Faecalibacillus	0.001304
Coprenecus	0.001217
Eggerthella	0.001098
Holdemanella	0.000859
Niameybacter	0.000859
Flavonifractor	0.000773
Pseudomonas	0.000708
Bombilactobacillus	0.000707
Lactococcus	0.000671
Actinomyces	0.000588
Pantoea	0.000583
Cellulosilyticum	0.000569
Ligilactobacillus	0.000471
Gilliamella	0.000414
Clostridium	0.000405
Lactiplantibacillus	0.000295
Varibaculum	0.000277
Bifidobacterium	0.00027
Eubacterium	0.0002
Leuconostoc	0.000191
Fusobacterium	0.000188
Lactococcus	0.000171
Desulfovibrio	0.000161
Cryptobacteroides	0.000112
Ellagibacter	8.03248E-05
Frischella	7.55278E-05
Eubacterium	6.02868E-05

Propionispira	5.86995E-05
Bilophila	5.8418E-05
Agathobacter	5.21008E-05
Apilactobacillus	5.21008E-05
Mesorhizobium	4.46578E-05
Weissella	4.2721E-05
Christensenella	3.72148E-05
Gemella	3.6618E-05
Fimenecus	3.65113E-05
Duncaniella	3.65113E-05
Luxibacter	3.65113E-05
Peptoniphilus	2.97719E-05
Lawsonibacter	2.9209E-05
Sporobacter	2.9209E-05
Barnesiella	2.9209E-05
Bartonella	2.23289E-05
Gemmiger	2.19068E-05
Alistipes	2.19068E-05
Dysosmobacter	1.48859E-05
Massilioclostridium	1.46045E-05
Neisseria	1.46045E-05
Finegoldia	1.2206E-05

SUPPLEMENTARY TABLE 6 Foods used in the simulations and their respective description from the Virtual Metabolic Human database.

Food group	Food	VMH Description
Vegetables	Broccoli	Broccoli, cooked, boiled, drained, without salt
	Brussel	Brussels sprouts, cooked, boiled, drained, without salt
	Cabbage	Cabbage, red, cooked, boiled, drained, without salt
	Carrot	Carrots, cooked, boiled, drained, without salt
	Cauliflower	Cauliflower, cooked, boiled, drained, without salt
	Celery	Celery, cooked, boiled, drained, without salt
	Cucumber	Cucumber, with peel, raw
	Eggplant	Eggplant, cooked, boiled, drained, without salt
	Green beans	Beans, snap, green, cooked, boiled, drained, without salt
	Green capsicum	Peppers, sweet, green, cooked, boiled, drained, without salt
	Lettuce	Lettuce, butterhead (includes boston and bibb types), raw
	Mushroom	Mushrooms, white, cooked, boiled, drained, without salt
	Onion	Onions, cooked, boiled, drained, without salt
	Pak choi	Cabbage, chinese (pak-choi), cooked, boiled, drained, without salt
	Potato	Potatoes, boiled, cooked in skin, flesh, without salt
	Pumpkin	Pumpkin, cooked, boiled, drained, without salt
	Sweetcorn	Corn, sweet, yellow, cooked, boiled, drained, without salt
	Spinach	Spinach, cooked, boiled, drained, without salt
	Squash	Squash, winter, butternut, cooked, baked, without salt
	Sweet potato	Sweet potato, cooked, boiled, without skin
	Tomato	Tomatoes, red, ripe, raw, year round average
	Yam	Yam, cooked, boiled, drained, or baked, without salt
	Zucchini	Squash, summer, zucchini, includes skin, cooked, boiled, drained, without salt
Fruits	Apple	Apples, raw, gala, with skin
	Banana	Bananas, raw
	Blackcurrant	Currants, european black, raw
	Blueberries	Blueberries, raw
	Cherry	Cherries, sweet, raw
	Feijoa	Feijoa, raw
	Gold kiwifruit	Kiwifruit, ZESPRI SunGold, raw
	Grape	Grapes, red or green (European type, such as Thompson seedless), raw
	Grapefruit	Grapefruit, raw, pink and red, all areas
	Green kiwifruit	Kiwifruit, green, raw
	Mandarin	Tangerines, (mandarin oranges), raw
	Mango	Mangos, raw
	Melon	Melons, cantaloupe, raw
	Nectarine	Nectarines, raw
	Orange	Oranges, raw, navels
	Peache	Peaches, yellow, raw
	Pear	Pears, raw, bartlett
	Pineapple	Pineapple, raw, traditional varieties
	Plum	Plums, raw
	Raspberries	Raspberries, raw
	Strawberries	Strawberries, raw
Cereals and starches	Barley	Barley, pearled, cooked
	Barley cereal	Babyfood, cereal, barley, prepared with whole milk
	Couscous	Couscous, cooked

	Noodles	Noodles, egg, unenriched, cooked, without added salt
	Oat cereal	Babyfood, cereal, oatmeal, prepared with whole milk
	Pasta	Pasta, cooked, unenriched, without added salt
	Rice	Rice, white, long-grain, regular, enriched, cooked
	Rice cereal	Babyfood, cereal, rice, prepared with whole milk
	Tapioca pudding	Puddings, tapioca, dry mix, prepared with whole milk
	White bread	Bread, white, commercially prepared (includes soft bread crumbs)
	Wholegrain bread	Bread, whole-wheat, commercially prepared
Dairy, eggs, and plant-based alternatives	Cottage cheese	Cheese, cottage, lowfat, 2% milkfat
	Eggs	Egg, whole, cooked, hard-boiled
	Mozzarella cheese	Cheese, mozzarella, whole milk
	Soymilk	Soymilk, original and vanilla, unfortified
	Tofu	Tofu, raw, regular, prepared with calcium sulfate
	Whole milk	Milk, whole, 3.25% milkfat, with added vitamin D
	Yoghurt	Yogurt, plain, whole milk, 8 grams protein per 8 ounce
Meats	Beef	Babyfood, meat, beef, strained
	Chicken	Babyfood, meat, chicken, strained
	Codfish	Fish, lingcod, cooked, dry heat
	Lamb	Babyfood, meat, lamb, strained
	Mackerel	Fish, mackerel, Atlantic, cooked, dry heat
	Mussels	Mollusks, mussel, blue, cooked, moist heat
	Pork	Babyfood, meat, pork, strained
	Salmon	Fish, salmon, Atlantic, farmed, cooked, dry heat
	Shrimp	Crustaceans, shrimp, cooked (not previously frozen)
	Tuna	Fish, tuna, white, canned in water, without salt, drained solids
	Turkey	Babyfood, meat, turkey, strained
Legumes, nuts, and seeds	Almond	Nuts, almonds, dry roasted, without salt add
	Black beans	Beans, black, mature seeds, cooked, boiled, without salt
	Cashew	Nuts, cashew nuts, dry roasted, without salt added
	Chia	Seeds, chia seeds, dried
	Chickpea	Chickpeas (garbanzo beans, bengal gram), mature seeds, cooked, boiled, without salt
	Green peas	Peas, green, cooked, boiled, drained, without salt
	Hazelnut	Nuts, hazelnuts or filberts, dry roasted, without salt added
	Lentils	Lentils, mature seeds, cooked, boiled, without salt
	Peanut	Peanuts, all types, dry-roasted, without salt
	Pecans	Nuts, pecans, dry roasted, without salt added
	Pumpkin seed	Seeds, pumpkin and squash seeds, whole, roasted, without salt
	Red beans	Beans, kidney, red, mature seeds, cooked, boiled, without salt
	Soybean	Soybeans, mature cooked, boiled, without salt
	Split peas	Peas, split, mature seeds, cooked, boiled, without salt
	Sunflower seed	Seeds, sunflower seed kernels, dry roasted, without salt
	White beans	Beans, white, mature seeds, cooked, boiled, without salt
Controls	Breastmilk	Milk, human, mature, fluid
	Infant formula	Infant formula, NESTLE, GOOD START SUPREME, with iron, ready-to-feed
